# The Neurobiology and Psychology of Pedophilia: Recent Advances and Challenges

**DOI:** 10.3389/fnhum.2015.00344

**Published:** 2015-06-24

**Authors:** Gilian Tenbergen, Matthias Wittfoth, Helge Frieling, Jorge Ponseti, Martin Walter, Henrik Walter, Klaus M. Beier, Boris Schiffer, Tillmann H. C. Kruger

**Affiliations:** ^1^Division of Clinical Psychology and Sexual Medicine, Department of Psychiatry, Social Psychiatry and Psychotherapy, Hannover Medical School, Hannover, Germany; ^2^Laboratory for Molecular Neuroscience, Department of Psychiatry, Social Psychiatry, and Psychotherapy, Hannover Medical School, Hannover, Germany; ^3^Department of Sexual Medicine, University Hospital Schleswig-Holstein, Kiel, Germany; ^4^Clinical Affective Neuroimaging Laboratory, Medical Faculty University Hospital Magdeburg, Magdeburg, Germany; ^5^Division of Mind and Brain Research, Charité – University Clinic Berlin, Berlin, Germany; ^6^Institute of Sexology and Sexual Medicine, Charité – University Clinic Berlin, Berlin, Germany; ^7^Division of Forensic Psychiatry, Department of Psychiatry, Psychotherapy, and Preventive Medicine, LWL-University Hospital Bochum, Bochum, Germany

**Keywords:** pedophilia, child sexual abuse, functional and structural MRI, neuropsychology, neurodevelopment, etiology, epigenetic, neurobiology

## Abstract

A pedophilic disorder is recognized for its impairment to the individual and for the harm it may cause to others. Pedophilia is often considered a side issue and research into the nature of pedophilia is delayed in comparison to research into other psychiatric disorders. However, with the increasing use of neuroimaging techniques, such as functional and structural magnetic resonance imaging (sMRI, fMRI), together with neuropsychological studies, we are increasing our knowledge of predisposing and accompanying factors contributing to pedophilia development. At the same time, we are faced with methodological challenges, such as group differences between studies, including age, intelligence, and comorbidities, together with a lack of careful assessment and control of child sexual abuse. Having this in mind, this review highlights the most important studies investigating pedophilia, with a strong emphasis on (neuro-) biological studies, combined with a brief explanation of research into normal human sexuality. We focus on some of the recent theories on the etiology of pedophilia such as the concept of a general neurodevelopmental disorder and/or alterations of structure and function in frontal, temporal, and limbic brain areas. With this approach, we aim to not only provide an update and overview but also a framework for future research and to address one of the most significant questions of how pedophilia may be explained by neurobiological and developmental alterations.

## Introduction

In the light of frightening and emotionally disturbing sexual offenses against children, experts have focused more on the level of sexual behavior, referred to subsequently as “offenses,” while not differentiating the causes for that behavior in an appropriate and adequate way. Concerning sexual offending against children, two groups can be distinguished: first, those who show no sexual preference disorder, but whom, for various reasons, sexually abuse children. Reasons include sexually inexperienced adolescents, mentally retarded persons, and those with antisocial personality disorders (ASPDs), or perpetrators within general traumatizing family constellations, which seek surrogate partners in children (Rice and Harris, 2002; Greenberg et al., 2005). These individuals are most likely diagnosed with various impulse-control disorders, accounting for their engaging in child sexual abuse (CSA) without a specific sexual preference for prepubescent children (Allnutt et al., 1996; Greenberg et al., 2005). Second, there are those who do display a sexual preference disorder, namely pedophilia (i.e., the sexual preference for prepubescent minors) and/or hebephilia (i.e., the sexual preference for pubescent minors) (Seto et al., 1999).

Although this preference increases the risk of engaging in CSA, only about 50% of all individuals who do sexually abuse children are pedophilic (Blanchard et al., 2001; Schaefer et al., 2010) and not every pedophilic individual actually has abused children. The other 50% of individuals that have abused children are those who do so without a sexual attraction to children; i.e., they lack the necessary social skills to develop and maintain emotional and sexual relationships with appropriately aged peers and look to “replacement partners” in children as a kind of “surrogate” (Beier, 1998; Seto, 2008; Mokros et al., 2012b).

Overall, there is great consensus regarding the negative consequences for victimized children. The number of known cases of CSA in Germany, for example, was on average 14,600 in the last 5 years (Bundeskriminalamt, 2012). The estimated incidence is far greater than what is reported to authorities. In the United States, the National Center for Victims of Crimes, as reported in Finkelhor et al. (2009), states that one in 5 girls and one in 20 boys are victims of CSA (Crimes, 2012). Additionally, CSA has important economical aspects. The estimated burden for the particular countries is enormous, including childhood health care costs, adult medical costs, productivity losses, criminal justice costs, and special education costs, which results in an estimated lifetime cost per victim of non-fatal child maltreatment in general of $210,000 in the USA (Fang et al., 2012).

In this article, we discuss pedophilia with a focus on recent findings of the definition, neuropsychology, and neurobiology (including neuroimaging) of pedophilia as a specific phenotype within the spectrum of human sexual preference. For that purpose, the article highlights the current gaps in literature and offers suggestions as to where the field of pedophilia research should head in order to close these gaps.

## Pedophilia and Pedophilic Disorder: A Psychological Perspective

### Classification of pedophilia

Pedophilia is defined as an ongoing sexual attraction toward pre-pubertal children (Freund, 1963, 1967; Seto, 2009). In the new DSM-5, pedophilia is de-pathologized by differentiating between the sexual preference for prepubescent children (i.e., pedophilia) and the disorder in case of additional factors. These factors include experiencing significant distress and impairment by fantasies and urges, or the acting out on behavioral level, including child pornography consumption and/or committing hands-on CSA offenses.

The estimated prevalence leads to questions about the diagnostic validity and reliability of pedophilia as a classification entity. According to the DSM-5, pedophilic sexual preference and the pedophilic disorder must be differentiated. As can be seen in Table 1, the behavioral criterion was not included in the DSM-5 as a specifier, though it holds relevance for researchers and clinicians. From a clinical point of view, both child pornography consumption and/or hands-on CSA offenses would count as preference behaviors (Seto, 2010; First, 2011).

**Table 1:** **Diagnostic criteria of a pedophilic disorder according to DSM-5**.

DSM-5 pedophilic disorder	
Over a period of at least 6 months, recurrent, intense sexually arousing fantasies, sexual urges, or behaviors involving sexual activity with a prepubescent child or children (generally age 13 years or younger)	**Specify if:** Sexually attracted to males Sexually attracted to females Sexually attracted to both
The fantasies, sexual urges, or behaviors cause clinically significant distress or impairment in social, occupational, or other important areas of functioning	**Specify if:** Limited to Incest
The person is at least age 16 years and at least 5 years older than the child or children in Criterion A	**Specify type:** Exclusive type (attracted only to children) Non-exclusive type

From a clinical perspective, it is necessary to stress that there are pedophilic men who restrict their desire for sexual contact with children to fantasies only, and other men who are at risk to commit an offense because fantasy alone does not satisfy their sexual desire. This second group is potential offenders who wish to reduce their increasingly overwhelming impulses with therapeutic help (Beier et al., 2009a,b; Schaefer et al., 2010; Wakefield, 2012). It is possible for these men to be diagnosed with Pedophilic Disorder – due to experiencing interpersonal distress – without them committing an offense.

The other group of pedophilic men includes those who have committed sexual offenses against children. These individuals may feel remorse (and seek help to avoid a relapse), while others do not. Note that both fulfill criterion B of the DSM-5, as shown in Table 1, means that it has to be diagnosed as Pedophilic Disorder. Furthermore, it is necessary to distinguish between the exclusive type of pedophilia (attracted only to children) and non-exclusive type, and whether the person is attracted to males, attracted to females, or to both.

It is a completely different situation for perpetrators who committed sexual offenses against children, which were not caused by a pedophilic preference. Those are the surrogate types of sex offenders and can be diagnosed within the category of impulse-control disorder, accounting for the lack of a sexual preference for children but the committed act of CSA (DSM-5: 312.89; ICD-10: 63.8). Moreover, most sexual assaults happen in the “Dunkelfeld” for approximately every reported case of CSA; another five are left unreported, suggest some scholars (Hall and Hall, 2007; Seto, 2009). Dunkelfeld is a German word that literally translates to “dark field.”

It is of great importance for clinical diagnosis whether or not an erotic preference for the body scheme of children on the fantasy-level exists. There is a high chance that this information would be given voluntarily by self-referred, self-motivated pedophilic men, but less likely by those who are already involved with the legal system (probation etc.). It is therefore essential for the assessment and a reliable diagnosis to obtain a cooperation/compliance level. In self-motivated pedophiles, this collaboration is highest and makes them a highly interesting target group for research (see Section “Methods for Diagnosing Pedophilia”).

This underlines that pedophilia as a sexual preference must be seen independently from sexual offending against children – otherwise there would be only offending pedophiles. From a research point of view, it is imperative to understand in what way the neurobiological conditions – notwithstanding sexual preference – encourage the sexual behavior. These are possibly the same mechanisms that also encourage offense-like behavior in men with other sexual preferences (for instance in the case of rape on the background of sexual preference for adult women). Additionally, research efforts have to unravel which neurobiological mechanisms determine and regulate sexual preference, and how preference and behavior are interconnected.

In the research domain, pedophilia is currently viewed as a phenotype of sexual preference within the realm of human sexuality, including various different phenotypes (e.g., the sexual orientation toward the same gender), only that it concerns a preferred age in addition to gender (Beier et al., 2009a,b; Schaefer et al., 2010). This is separate from, but in addition to, behavioral manifestations including the use of child pornography and the commitment of child sexual offenses (Beier et al., 2009a,b; Neutze et al., 2011). Consequently, the sexual preference itself cannot be considered a mental disorder similar to how a homosexual orientation was considered in the 1970s in the United States of America (Green, 2002). Separating sexual preference from psychosocial impairment, thus allowing for the practice of various sexual behaviors with consenting partners, has been applied within the new DSM-5 with the other paraphilias as well, including fetishism, bondage/dominance-sadism/masochism, and is therefore not specific to pedophilia (Wright, 2010, 2014).

### Epidemiology of pedophilia

The most commonly asked question about pedophilia is how frequently it occurs. Obtaining reliable incidence numbers of pedophilia as a preference disorder is difficult as individuals are typically unwilling to admit pedophilic preferences, particularly when offenses have been committed. The prevalence of a true pedophilic sexual preference is approximately 1%, but when general fantasies are investigated, that prevalence can reach up to 5% among men in the general population, extrapolated from the studies discussed below.

Some studies suggest that the prevelance of pedophilia may be between 3% and 5% in the general population (as reviewed by Seto, 2009). In penile plethysmography studies of men with sexual offense histories against children, these prevalences can jump from 30% for men with one offense to 61% for men with 3 or more sexual offenses against children (Blanchard, 2010; Seto, 2009).

A first population-based study concerning this issue was based on the Berlin Male Study (BMS), in which the prevalence of erectile dysfunction, its age-dependency, and its relation to general health variables as well as quality-of-life measures were determined in 6000 men, aged 40–79 (Schäefer et al., 2003; Englert et al., 2007). A total of 1915 men took part in the first phase of this study. These men were then invited to further participate in a comprehensive sexological study by responding to an extensive questionnaire on sexual experiences and behavior. The outcome was a sample of 373 men, of whom 63 were single and 310 were in a relationship. Fifty-seven percent of the questioned men recognized at least one paraphilia-associated arousal pattern as part of their fantasies, 46.9% of this group used them for arousal enhancement during masturbation, and 43.7% acted out these patterns in a relationship. The finding of relevance here is that 3.8% acted out a pedophilic preference on the behavioral level – which means of these men – 14 men acted out their impulses toward children. Taking these 14 cases into account, the prevalence of a pedophilic sexual preference in Germany can be extrapolated to approximately 3.8% in the worst case (calculated based on the selected sample of 373 men) (Ahlers et al., 2011). However, pedophilia was not strictly assessed in this sample; thus, this prevalence should be interpreted with caution.

Much higher is the prevalence in an older anonymous self-report survey study of 193 healthy male college students: 21% admitted some degree of sexual interest in children, 9% admitted to having sexual fantasies involving children, 5% admitted to masturbating to orgasm through these fantasies, and 9% admitted that they would have sex with a child, if it were guaranteed they would never be caught (Briere and Runtz, 1989). Yet, it is important to note that this study did not specifically investigate the preference of pedophilia, rather sampled fantasy.

Considering the lack of reliable estimates of the prevalence of pedophilia in general, prevalence estimates for the subtypes of pedophilia also remain scarce. The current estimated prevalence of homosexual pedophilia is anywhere between 9 and 40% (Hall and Hall, 2007); the ratio of heterosexual to homosexual pedophiles was approximately 1.4:1 among men with CSA offenses in another study using phallometry (Freund and Watson, 1992). Prevalence estimates of bisexual pedophilia are not yet available due to measurement complexity (Hall and Hall, 2007).

Although pedophilia is generally regarded as a phenomenon in males (Seto, 2008), victim surveys show that a female perpetrator was indicated by between 14 and 24% of sexually abused males and by between 6 and 14% of sexually abused females (Green, 1999). In a Dutch report (Wijlman et al., 2010) investigating female sex offenders in the Netherlands between 1994 and 2005, common characteristics included intellectual impairment, a high current and/or lifetime prevalence of psychiatric or personality disorders, and a high lifetime prevalence of neglect and sexual abuse. Frequently, the abuse against a child is carried out in collaboration with a male partner or victims are seen as surrogates to replace less than desirable relationships. Currently, there is no reliable estimate of pedophilia in women and the question remains whether pedophilia, as currently defined, even exists in women.

### Methods for diagnosing pedophilia

The most important method for ascertaining the phenotype of sexual preference is the clinical exploration. In this process, the content of sexual fantasies during masturbation is particularly significant as it reveals gender preferences, body scheme age of the “partner,” and favored practices. Here, it is possible to assess the sexual preference structure in detail including the differentiation between exclusive and non-exclusive types of pedophilia and hebephilia (Beier et al., 2013). If the legal system is involved, the patient might not (or only partially) cooperate due to the possibility of new accusations.

**Figure 1:**
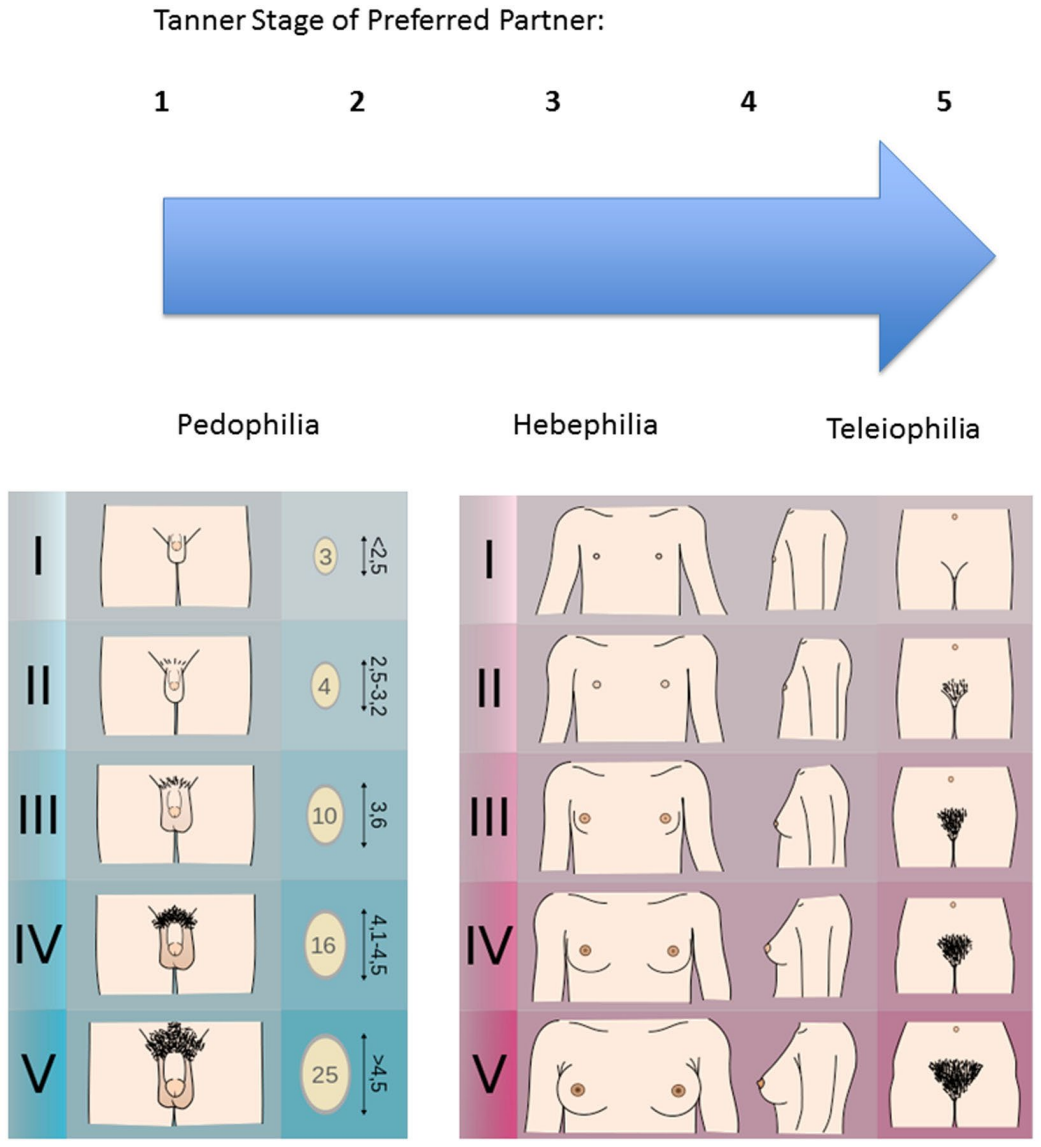
**Tanner scales of males and females as used in sexual preference assessment**. Image credit: Michał Komorniczak, 2009, CC-BY-SA. Tanner Scale Male: http://goo.gl/7cxTLM. Tanner Scale Female: http://goo.gl/haB9Cb, both accessed June 09, 2015.

For assessment of body scheme age preference, the five Tanner stages have proven useful. They describe the process of physiological maturing by focusing on the development of the secondary sex characteristics from 1 (prepubescent) to 5 (adult). Therefore, Tanner stage 1 concerns the prepubescent developmental phase, displaying a complete lack of secondary sex characteristics showing no facial or pubic hair, no penile or scrotal enlargement in males, no breast development or pubic hair growth in females. Tanner stage 2 corresponds to the onset of breast budding in females and testicular enlargement in males. Tanner stage 3 depicts the breast and areola development in females, continued testicular growth and initial penile lengthening in males. Tanner stage 4 corresponds to increased breast and areola growth, initial separation from surrounding breast tissue in females, and in males testicular volume increases, scrotum darkens, penile elongation continues. Tanner stage 5 represents full maturity, complete breast development, and separation from surrounding breast tissue in females, full penile growth and scrotum darkness, and testicular volume in males, and full pubic hair coverage in both (Marshall and Tanner, 1969, 1970). Please refer to Figure 1 for a visual explanation of Tanner stages and their relationship to sexual preference. The Tanner stages can be very useful during the exploration of sexual preference and are an essential component of the diagnostic procedure in various treatment and research programs (Seto, 2008). Pedophilia is here defined as the erotic attraction to a prepubescent body scheme corresponding to Tanner stages 1 and 2 (Blanchard, 2010).

Child pornography use is also strongly related to pedophilia. As a study deriving from the German Dunkelfeld Prevention Project concluded, among 345 pedophiles admitting one or more sexual offenses against children, 37% have solely used child pornography, 21% committed exclusively hands-on sexual contacts with a minor, and 42% have committed both (Neutze et al., 2012).

The most well-known objective method of measuring pedophilic interest is penile plethysmography (PPG) or phallometry. This method measures genital sexual arousal through sexual stimuli and is based on the relative change in penile response. Sexual preference can be determined as the relative change in penile response to various classes of sexual stimuli (according to Tanner scales), such as prepubescent, pubescent, or adult, female, or male targets. At least one of these classes should correspond to the individual’s stated or inferred sexual preference. There are two different methods of phallometry, circumferential and volumetric. The circumferential method measures intra-individual changes in penile girth through a wire band fitted around the base of the penis in response to differing classes of sexual stimuli (Bancroft et al., 1966). The volumetric method uses a glass tube fitted around the penis to measure calibrated air output as a result of erection (Freund, 1963). The latter method is sensitive to small changes, making it useful when assessing partial- or non-admitters, or pedophilic men that attempt to hide their sexual preferences (Freund and Blanchard, 1989; Blanchard et al., 2001). Both measures are valid and reliable, producing sensitivities between 55 and 61% and specificities between 95 and 96% (Kuban et al., 1999; Blanchard et al., 2001; Hughes, 2007).

Self-reported interest in children, child pornography use, and the number of children as sexual victims all uniquely contribute to phallometrically assessed sexual interest in children (Mokros et al., 2012b). Additionally, child pornography users showed a greater phallometric response to sexual child stimuli than non-pedophilic child sexual offenders (CSO), and there was no significant difference within the child pornography group between those who had committed sexual offenses against children and those who had not (Seto et al., 2006).

While phallometry has long been the ‘gold standard’ in assessing sexual preferences, other methods have been developed using indirect and implicit tests to cope with faking responses. One of the more strongly validated tests is the viewing time paradigm measuring the length of time a participant spends looking at specific images as an indicator for sexual preference. Research assumes that all participants, including CSO, will look significantly longer at sexually arousing stimuli (Mokros et al., 2012a). An initial study by Abel showed a high specificity and sensitivity to classifying sexual offenders against adolescent boys (98% control vs. 90% offender), only moderate sensitivity for those against boys under 14 (98 vs. 76%), and lower performance against adolescent/young girls (77 vs. 91%) (Abel et al., 1994). A follow-up study found that between the viewing time paradigm and the PPG, discriminatory capacity was negligible, showing no significant differences in their abilities to discriminate among sex offenders with deviant sexual interest in adolescent females, adolescent males, female children, or male children (Abel et al., 1998). Abel et al. (1998) suggested, however, that the PPG may be slightly better at classifying offenders against young boys, although this claim needs urgent replication.

The pictorial Stroop was developed as a modified, sexual version of the original Stroop task, measuring implicit sexual associations that exert their effects automatically, which are difficult if not impossible to control consciously. Research supports its use among a sample of CSO (*n* = 24) compared to controls (*n* = 24), with those admitting deviant sexual interest in children having the greatest mean bias for child stimuli as compared to adult stimuli (partial η^2^ = 0.07) (Ciardha and Gormley, 2012). However, other factors could have contributed to the results, justifying a need for further refinement. Results in a separate study of 35 men, 11 of who were homosexual and 24 were heterosexual, reporting no history of child sexual offenses indicated a discriminatory ability between heterosexual men and homosexual men using female stimuli, but could not discriminate among preferred ages. The authors suggested that other mechanisms are responsible for rating child stimuli, thus decreasing the validity for this test among pedophilic participants (Bourke and Gormley, 2012).

Eye tracking and pupil dilation may also indicate sexual preference and results show that men react more strongly in these studies than women. Heterosexual men did initially orient to their stated preference and eye fixations were significantly longer than when looking at non-preferred stimuli (Fromberger et al., 2012b). In a study investigating pedophilia, eye tracking produced high sensitivity and specificity, 86.4 and 90.0%, respectively (Fromberger et al., 2012a). Heterosexual women reacted similarly to stimuli of both sexes, whereas heterosexual men, homosexual men, and homosexual women reacted most strongly to their stated partner gender in pupil dilation research (Rieger and Savin-Williams, 2012). However, criticisms have been put forward suggesting that the success seen in heterosexual and homosexual participants to respective stimuli in pupil dilation studies is attributable to factors other than sexual preference, such as luminance, salience of the stimuli, and emotional reaction (Beatty and Lucero-Wagoner, 2000; Rieger and Savin-Williams, 2012). These methods have not yet been used in the sexual age preference measurement of pedophiles, but do hold promise as collateral information for diagnosis.

The aforementioned methods are not without their flaws, such as test–retest reliabilities or the ability to fake results. These methodological differences have led to interest of using specific functional Magnetic Resonance Imaging (fMRI) techniques in order to classify pedophilic interest (Ponseti et al., 2012). Results have shown that a preference-specific BOLD pattern is evident, which can be potentially used as a diagnostic tool. Keeping these findings in mind, this methodology could be used in the future as a classification paradigm.

### Co-morbidities with pedophilia

Pedophilia does not always occur in isolation; men with pedophilia often have extensive histories of psychiatric disorders that, in extreme cases, can overshadow discovery of etiological course. Whether this is a secondary phenomenon that relates to emotional and social consequences of this preference, or whether these are true co-morbidities remains elusive.

Kramer (2011) addresses a point that currently many pedophilia researchers are facing: should we continue to classify pedophilia as a separate psychiatric disorder or as a sexual orientation, when patients harbor complaints not only of the preference but of the pressure under which they suffer? This pressure often precedes the onset of psychiatric illness (most often mood or anxiety disorders), which then precedes the decision to seek psychiatric help (Kramer, 2011). Due to a temporal-causal relationship being nearly impossible to determine in these cases, the DSM-5 has differentiated among those who experience the sexual preference but do not suffer and those who do, leading us back to Pedophilia vs. Pedophilic Disorder, regardless of whether or not child sexual offenses have occurred (Kramer, 2011).

A relationship has been identified between pedophilia and co-morbid psychiatric disorders. Among pedophiles in residential or outpatient treatment, two-thirds had a lifetime history of mood or anxiety disorders, 60% had lifetime substance abuse history, with 51% naming alcohol as their drug of choice, and 60% qualified for a personality disorder diagnosis of which obsessive-compulsive (25%), antisocial (22.5%), narcissistic (20%), and avoidant (20%) were most common, as reported in reviews (Fagan et al., 2002; Green, 2002). Kalichman (1991) investigated 144 sexual offenders divided into child, adolescent, and adult offenders (although not controlled for pedophilic preference) for state and trait measures of anger, anxiety, self-esteem, and various measures on the Minnesota Multiphasic Personality Inventory (MMPI). Findings demonstrated that child offenders, as compared to offenders against adolescents and offenders against adults, scored significantly higher on 2 out of 3 scales for the “neurotic” triad (hypochondriasis and hysteria), and 3 out of 4 scales for the “psychotic tetrad” (paranoia, psychasthenia, schizophrenia), and were significantly more introverted (Kalichman, 1991). These findings suggest that child sexual offending is characterized by emotional disturbance and higher rates of psychopathology. Moreover, these findings do not necessarily mean that there is a direct connection to pedophilia.

In an empirical study comparing 20 forensic inpatients with pedophilia attracted to males or attracted to females (but lacking information of whether they were exclusive or non-exclusive types, respectively) and 24 matched male controls on various psychiatric measures, findings included increased personality subscale scores from the MMPI-2 for psychopathy and paranoia, with enhanced scores for hypochondriasis, depression, hysteria, and masculinity/femininity, psychasthenia, schizophrenia, and social introversion (Kruger and Schiffer, 2011). Furthermore, that study shows that 61.1% of the sample qualified for a personality disorder diagnosis, with Borderline Personality Disorder (22%; from Cluster B) and Avoidant Personality Disorder (33%; from Cluster C) as the two most common (Kruger and Schiffer, 2011). Self-report results in low socioeconomic status individuals, often including non-pedophilic sexual offenders against children and rapists, indicate more social anxiety, less social poise, and a decreased ability to appropriately socially assert oneself, relating to the cognitive distortions seen among these groups of negative attitudes toward women, reinforcing beliefs about sex with children, denial of harm to victims, and misattribution of responsibility of offending (Geer et al., 2000).

These results indicate that delinquent pedophiles seem to differ from healthy controls (HC) for Axis I and II psychiatric comorbidity development. However, it remains to be further explored whether these alterations are linked specifically to the sexual preference of pedophilia or to the commitment of sexual offenses against children, as meant by the behavioral criterion of Pedophilic Disorder (impaired control over sexual impulses) or a combination of both.

### Neuropsychological findings associated with pedophilia

Aside from psychiatric comorbidities, neuropsychological alterations are another important issue, which have been addressed by several studies, and may further contribute to the understanding of the development and course of pedophilia. The majority of studies in the following paragraphs were conducted as uncontrolled studies, mostly using incarcerated CSO, which were not carefully screened for incarceration stress or for pedophilic sexual preference. Therefore, the results are not generalizable and need careful consideration.

Initial studies exploring the neuropsychological correlates of pedophilia often used uncontrolled designs with incarcerated pedophilic men. An initial study by Tarter et al. (1983) among recently incarcerated adolescent offenders and controls, no neuropsychological differences were found among the groups on the Wechsler Adult Intelligence Scale (WAIS) or Pittsburgh Initial Neuropsychological Test System. Furthermore, among incarcerated adult male sex and non-sex non-violent offenders, no differences were seen in any neuropsychological test variables, after age and education status were accounted for (Abracen et al., 1991). Keeping in mind that the subject groups were incarcerated at the time of study and that pedophilia was not explicitly examined, the results are limited in their generalizability and specificity to pedophilia.

In another study, heterosexual and homosexual pedophiles were tested, but each group, plus one control group, had sexual offense histories and were incarcerated at the time of the study. Fully admitting, heterosexual pedophiles had gender differentiation indices (or the erotic sensitivity for the gender-differentiating body shapes that distinguish physically mature males and females), which were greater than for non-admitting heterosexual pedophiles, but no differences were found for either homosexual or bisexual pedophiles. This highlights that fully admitting heterosexual pedophiles prefer the body shapes of female children, whereas the partial- or non-admitting pedophiles do not seem to discriminate between victim body types (Freund et al., 1991; Freund and Kuban, 1993). Gillespie and McKenzie (2000) investigated neuropsychological differences among forensically incarcerated sex offenders and non-sex offenders and found no significant differences on any of their measures, including the WAIS, Trail Making Task, List Learning test, Controlled Oral Word Association test, and National Adult Reading Task (NART). Among personality disordered offenders, violent sex offenders, violent non-sex offenders, and non-violent, non-sex offenders, no differences were noted on any neuropsychological test variables, including the WAIS, Trail Making Task, Face Recognition, Wechsler Memory Scale-Revised, and Wechsler Recognition Memory Task (Dolan et al., 2002). As with previous studies, neither incarceration status nor sexual preference was controlled for, which limits the generalizability results to pedophilic men.

Within a heterogeneous group of 20 sexual offenders (not differentiated by gender or age preference), executive functions were significantly impaired; however, the generalization of these findings to pedophilia is limited considering the nature of the group (Joyal et al., 2007). Findings included prolonged Stroop effects (reaction time differences between incongruent and congruent trials: 77.2 vs. 59.4 s) that were indicative of stronger interferences and impaired verbal skills, with deficits seen in verbal fluency and in verbal processing and memory. While response inhibition and sustained attention showed impairments, set shifting, cognitive flexibility, and visuo-spatial integration abilities were normal.

**Figure 2:**
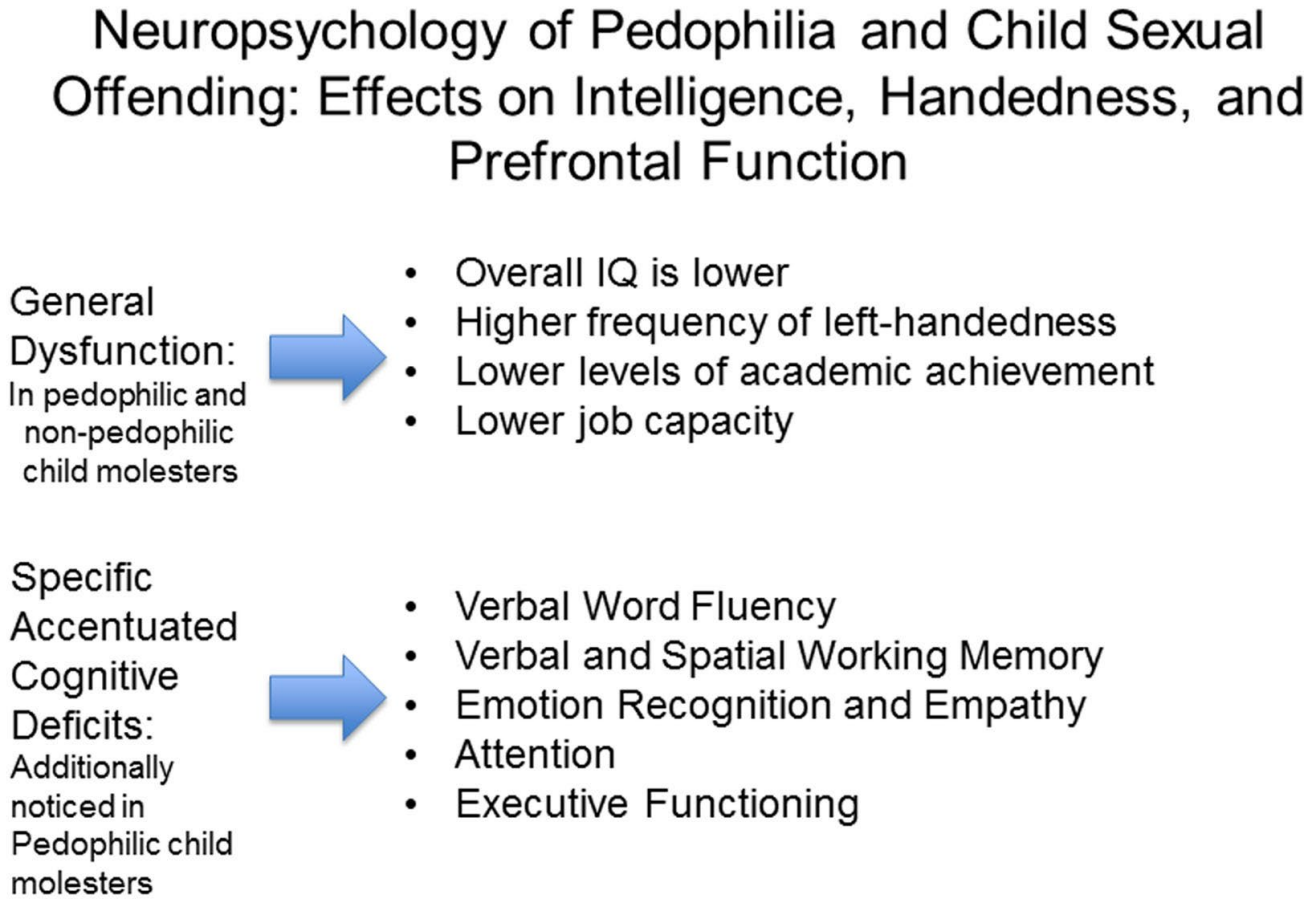
**Overview of neuropsychological findings in pedophilia and child sexual offending**.

In a more controlled study that differentiated among pedophilic CSO with a primary sexual interest in prepubescent children (*n* = 20) and non-pedophilic CSO with a primary sexual interest in adults (*n* = 20) and controls (*n* = 20), Suchy et al. (2009) investigated patterns of executive function. Non-pedophilic offenders showed general impairments, whereas among pedophilic offenders, deficits were more specific. Both groups showed deficits in executive functions, specifically showing slower information processing speeds for pedophilic offenders and semantic knowledge impairments in non-pedophiles (Suchy et al., 2009, 2014). Eastvold et al. (2011) corroborated these results, further specifying that although pedophilic (incarcerated) offenders (*n* = 30) do not show generalized executive functioning impairments, they instead show a distinct pattern of differences, not all of which are worse than that for control participants, characterized by impaired performance on behavioral inhibition measures (partial η^2^ = 0.129), but better performance in abstract reasoning measures and planning measures (partial η^2^ = 0.132) as compared to non-sexual offender (*n* = 29) and non-pedophilic sexual offender controls (*n* = 30). A further study also found specific deficits in inhibition in 15 pedophilic offenders as compared to non-sexual offenders and non-offender controls, whereas more global executive functioning impairments were seen in non-pedophilic child molesters (Schiffer and Vonlaufen, 2011). Yet, new findings are showing that pedophilic men are characterized by a distinct neurocognitive weakness, performing worse than controls on measures of behavioral inhibition and information processing (Suchy et al., 2014), but do not have a more planning-oriented response style as found by Eastvold and colleague (Habermeyer et al., 2013b). Please refer to Figure 2 for an overview of neuropsychological impairments seen in non-pedophilic and pedophilic child sex offenders.

Despite these results, further research has indicated contradictory results regarding executive functioning impairments. For example, heterosexual formerly incarcerated pedophiles scored lower than controls in the Wechsler Adult Intelligence Scale-Revised (WAIS-R) vocabulary subtest measuring verbal fluency, but performances on tests measuring impulsivity or attention capabilities were all normal: Wisconsin Card Sorting Task (WCST), Trail Making Task A and B, Gambling Test, Stroop Color-Word Test, and Controlled Oral Word Association test (Cohen et al., 2002). In a study using a shortened version of the WAIS-R known as the WIP, the WCST, the d2 Attention Deficit Test, and the Corsi Block-Tapping Test showed that – unlike the Cohen study (Cohen et al., 2002) – convicted pedophiles serving prison sentences in a forensic treatment facility had impaired performance on all subtests of the WIP except for completing images (Kruger and Schiffer, 2011). The participants consisted of both exclusive heterosexual and homosexual pedophiles. Significantly weaker performance on the d2 Attention-Deficit Test was also seen, but this difference disappeared once participant age was controlled. The Corsi Block-Tapping test and the WCST, however, showed normal performances among the pedophilic offender group. Cantor et al. (2005), in their meta-analysis of IQ data in sex offenders, found not only that lower IQ between 90 and 95 was associated with sexual offending against children and with pedophilia specifically, but also that the younger the victim, the lower the IQ of the offender.

These results suggest that disturbed and prosecuted pedophiles do show deficits in executive functioning, which might be due rather to mental disturbances and not to the sexual preference. This is in contrast to findings from the APSD/psychopathy literature that suggests, at least among community samples measured for psychopathic traits, that these personality traits are linked to deficits in response inhibition and impulsivity, specifically with social deviancy associated with overall deficits in executive functioning and response inhibition, whereas callous-unemotional traits observed in psychopathy are associated with improved executive functioning abilities (Sellbom and Verona, 2007). In ASPD, broad executive function domain deficits have been noted in response inhibition, planning, and rule acquisition, and reversal learning, suggesting that previous studies examining pedophilia may have been measuring ASPD or simply an incarceration stress effect in their incarcerated samples rather than pedophilia. This is in contrast to recent studies that have found processing speed impairments in pedophiles, but few other deficits suggestive more of offense status effects than sexual preference effects (Eastvold et al., 2011; Kruger and Schiffer, 2011; Schiffer and Vonlaufen, 2011; Suchy et al., 2014).

Future studies need to carefully control for psychiatric comorbidities, incarceration status, and offender status, as no research to date has examined neuropsychological deficits in potential offenders or non-offending pedophiles. Only with these studies will the true nature of neuropsychological impairment be illustrated.

## Neurobiology and Neurodevelopment of Pedophilia

### Introduction and conceptual framework

Research regarding the etiology of pedophilia suggests the view of a complex and multifactorial phenomenon in which the influences of genetics (Blanchard et al., 2007), stressful life events, specific learning processes (Jespersen et al., 2009a), as well as perturbations in the structural integrity of ‘pedophilic’ brains may generate this specific phenotype of a sexual preference (Schiffer et al., 2007; Schiltz et al., 2007; Cantor et al., 2008). Initial theories relied mainly upon psychological mechanisms to account for a pedophilic preference, including classical and operant conditioning, as the behavioral mechanism through which the ‘abused-abuser’ theory by Freund et al. (1990) and Freund and Kuban (1994) could be explained as well as attachment style in childhood as a marker for dysfunctional cognitive sexual schemas in adulthood (Beech and Mitchell, 2005).

The first theories to account for sexual behavior disorder associated with pedophilia suggested masturbatory conditioning [e.g., Laws and Marshall (1990)] or childhood sexual abuse (Freund et al., 1990; Fedoroff and Pinkus, 1996) as causal explanations. However, as Seto purports, due to lack of stringent methodology that includes proper control groups, small experimental or treatment effect sizes, and lacking knowledge of effect duration, these theories are not well supported. Beyond this, the majority of victims are female, whereas the majority of offenders are male, and if conditioning were the only logical theory to explain the etiology of pedophilia, it stands to reason that there would be more female pedophiles than are clinically seen (Seto, 2008; Jespersen et al., 2009b). However, a study by Klucken et al. (2009) showed that men are more easily conditioned through exposure to sexual stimuli than are women, casting significant doubt on the conditioning theory as it applies to female pedophiles. Currently, there is a strong push to understand the brain’s role in sexual preference development, particularly as it relates to pedophilia.

As discussed in a previous review by Seto (2008), there are three major neurobiological theories, which have come to be connected to pedophilia but all have the same shortcoming that they rely on data based on cases of pedophiles who have other psychological disorder diagnoses, are incarcerated or otherwise legally sanctioned, or are not sufficiently diagnostically classified (i.e., not differentiating between the exclusive or the non-exclusive type, etc.).

The first is the “frontal lobe” theory that refers to orbitofrontal and left and right dorsolateral prefrontal cortex differences that are often seen in pedophilic men (Graber et al., 1982; Flor-Henry et al., 1991; Burns and Swerdlow, 2003; Schiffer et al., 2007, 2008a,b). As the orbitofrontal cortex is responsible for behavior control (Bechara et al., 2000; O’Doherty et al., 2003), especially inhibiting sexual behavior, volume differences or dysfunction in this area may explain the sexual behavior disorder associated with pedophilia, although not pedophilic sexual preference.

The second major theory is the “temporal lobe” theory, referring to reports of hypersexuality accompanying pedophilia. Studies have shown that disturbances of the temporal lobes can result in an increase in pedophilic behaviors or an increase in the breadth of deviant sexual interests (Hucker et al., 1986; Langevin et al., 1988). These disturbances include temporal lesions and hippocampal sclerosis (Mendez et al., 2000). Ponseti noticed further differential temporal lobe activations in pedophilic men that highlight a hypersexuality-specific activation profile, further supporting the role of the temporal lobe in the expression of hypersexuality that is often seen with sexual behavior disorders (Schiltz et al., 2007; Ponseti et al., 2012). However, this theory also does not fully explain the etiology of the preference.

The third major neurobiological theory holds that differences in the sex dimorphic brain structures affected by the masculinization of the male brain would more strongly affect pedophilia development. Furthermore, the volumes of these structures would be influenced, but the hypothesis failed to state in what direction these changes occur, i.e., either increased or decreased volumes as a result of testosterone exposure. In the frontal and temporal lobes, these differences would be limited to those sexually dimorphic structures, rather than a generalized difference in region volume, but research has not supported the hypothesis (Cantor et al., 2008).

Furthermore, there is an additional theory that combines the frontal and temporal lobe theories. It states that the frontal and temporal lobes affect pedophilic sexual preference expression and its associated behaviors differently, with the frontal lobe (orbitofrontal and dorsolateral prefrontal cortices) accounting for committing the sexual offenses against children and the temporal lobe (amygdala and hippocampus) accounting for the sexual preoccupation with children often seen in pedophilic men (Seto, 2008, 2009; Poeppl et al., 2013).

Currently, pedophilia is often viewed as an interaction among neurodevelopmental factors based on genes and the (*in utero*-) environment as previously discussed (Becerra García, 2009). This theory holds that pedophilic sexual preference is a neurodevelopmental disorder corroborated by increased rates of non-right-handedness, shorter stature, lower intelligence, head injury, prenatal androgen levels, and the associated neuronal structural and functional differences that are present since childhood and/or adolescence. The exact directions of these relationships to pedophilic sexual preference, committing child sexual offenses, or consuming child pornography are still to be disentangled. There is currently no causal evidence yet to support a role in pedophilic sexual preference development.

### Neurodevelopmental correlates of pedophilia

The prevailing perspective among biologists was that sex differences are linked solely to the exposure to testosterone *in utero* [see Phoenix et al. (1959) and Ehrhardt and Meyer-Bahlburg (1979)]. The masculinization of an initially undifferentiated human female brain is caused by testosterone’s induction of organizational effects during a limited period of time, as extrapolated from animal research. Sexual differentiation and development of subsequent sexual preference are likely an interplay between the impact of sex chromosomes on gene expression and sex hormones (Bao and Swaab, 2010). In pedophilia, research investigating biological differences is underway and studies have already highlighted structural and functional differences. The following is a discussion of findings that are classified as neuropsychological; however, the onset of these differences is *in utero*, childhood, and adolescence, thus suggesting that these findings are actually a part of human development and contribute to pedophilic preference onset rather than acting as consequences thereof.

As a group consisting of primarily incarcerated individuals, pedophilic men show a doubled rate of head injuries before age 13, though after 14 years of age the difference is no longer significant, highlighting possible causative effects in multiple areas of cognitive functioning. While prenatal perturbations influence cognitive functioning and disorder development, so can head injuries resulting in unconsciousness in childhood, especially before age 13 years (Blanchard et al., 2002, 2003). This is a result of cortical development plasticity during childhood, when synaptic myelination and pruning are at their peak (Zhong et al., 2013). Of 725 originally tested, 685 pedophilic men participated in a study investigating the role of head injuries with associated loss of consciousness in pedophilia development. Pedophilic participants reported a significantly higher number of head injuries that resulted in a loss of consciousness prior to age 13 than did non-pedophilic child sexual offender participants. These results also positively correlated with a diagnosis of attention deficit-hyperactivity disorder and left-handedness among pedophilic participants.

More importantly, the more child victims each pedophile had correlated positively with each additional head injury before age 13, but not those sustained later in adolescence or adulthood (Blanchard et al., 2003). However, no studies have yet been conducted investigating head injuries in non-incarcerated pedophilic men with histories of CSA, or those with no such histories. Also lacking are studies on the prevalence of head injuries in children in general, as well as for the number of children with head injuries who subsequently go on to commit sexual offenses against children in adulthood.

The organizational–activational hypothesis was initially developed by Phoenix and his colleagues in the 1950s in consequence to observations that a temporary rise in prenatal and early post-natal testosterone shapes development by masculinizing and defeminizing neural networks in males, whereas the absence thereof results in the development of female-typical neural phenotype (Phoenix et al., 1959; Schulz et al., 2009). According to the organizational–activational hypothesis, pre- and perinatal as well as pubertal/adolescent androgens are able to shape cortical circuits (organization), whereas in adults androgens can only modulate the activity of these circuits (activation). The process of sexual differentiation occurs between weeks 12 and 18 of prenatal life and during the first 2 months after birth, periods during which testosterone has organizational effects on the brain. During this time, not only behavior is programed, depending on the level of exposure to testosterone, but also handedness, total digit length, and second to fourth finger length ratios (George, 1930; Rahman, 2005). These neuroendocrinological developmental differences are then activated during puberty and their relationship to pedophilia development will be discussed further in the coming paragraphs.

In understanding the relationship between testosterone, the brain, sexual behavior, and the rise of sexual deviancy, one must first understand how testosterone influences the brain. In vertebrates, androgen receptors (ARs) can be found in several brain regions, including the lateral septum, posteromedial bed nucleus of the stria terminalis (BNSTpm), medial preoptic nucleus of the hypothalamus, ventral premammillary nucleus, ventromedial nucleus of the hypothalamus, and the medial amygdaloid nucleus, otherwise found in the temporo-occipital, superior-parietal, and orbitofrontal cortices (Wood and Newman, 1999; Jordan et al., 2011a).

Research has shown a relationship between prenatal androgen exposure and hand preference in pedophilic men with a history of sexual offending against children. These men show a trend for increased rates of sinistrality – more efficient use of the left side/hand and is preferred – whereas hebephilic men show increased rates of ambiguous-handedness (Fazio et al., 2014) as compared to teleiophiles, and this has been discussed as an indicator of developmental perturbations resulting from a lack of prenatal testosterone exposure (Cantor et al., 2004). Homosexuality has also been associated with a higher prevalence of left-handedness (Cantor, 2012), and it would be of interest to see whether the higher prevalence of left-handedness seen among pedophilic men is attributable to pedophilia specifically or to a higher rate of homosexuality within this population as compared to teleiophilic men. More specifically, approximately 11% of the general non-offender population is non-right-handed, whereas pedophilic men with histories of sexually offending against children are approximately 15% non-righthanded, this difference being significant (Bogaert, 2001; Cantor et al., 2004, 2005; Blanchard et al., 2007; Rahman and Symeonides, 2008). Future studies should control for sexual orientation (homosexuality vs. heterosexuality) when examining handedness in pedophilia.

These effects also influence the second to fourth finger length ratio (D2:D4) (Voracek et al., 2007), a marker altered also in other psychiatric disorders including alcohol dependence (Lenz et al., 2012). The D2:D4 ratio is smaller in men than in women and is used as an indirect marker of prenatal testosterone exposure (Beaton et al., 2011). Additional differences in sexual orientation exist, such that the D2:D4 ratio is smaller in homosexual women compared to heterosexual women, as well as homosexual men compared to heterosexual men (Williams et al., 2000; Rahman and Wilson, 2003; Rahman, 2005; Manning et al., 2007). Although prenatal testosterone exposure affects both hand preference and D2:D4 ratio, the data here are equivocal and no firm conclusions have been drawn regarding the absolute relationship between hand preference and D2:D4. However, exposure to prenatal testosterone does not affect the D2:D4 ratio between 9 weeks gestation and birth, in contrast to hand preference, where differences are noted here and possibly after puberty (Lenz et al., 2012). How this applies to pedophilia is currently under investigation.

The following markers of neurodevelopmental abnormality have also been implicated in the neurodevelopmental processes contributing to pedophilia: sibling sex composition, maternal and paternal age at birth, and the fluctuating asymmetry of finger lengths and wrist widths. Pedophiles have a greater number of older brothers (Lalumière et al., 1998; Côté et al., 2002). Greater paternal age at birth was related to an increased chance of homosexuality, whereas greater maternal age increased risk for pedophilia, specifically (rather than generalized paraphilia) (Rahman and Symeonides, 2008).

Considering the effects of neurodevelopmental perturbations and executive functioning on pedophilia development, it seems worthwhile to consider the effect of intelligence. Research results have been contradictory: for example, generalized sexual delinquency is related to lower intelligence, whereas among groups of non-sexual offenders, pedophiles, and non-pedophiles, neither education level nor intelligence differed significantly. However, when pedophilic participants were separated by use of child pornography, those who had no history of child pornography use showed a decreased IQ and lower mean education level as compared to those who did (Briken et al., 2006; Blanchard et al., 2007; Schiffer and Vonlaufen, 2011). The main caveat to this research is that child pornography is considered a reliable indicator of pedophilic sexual interest, therefore confounding any results found with education or intelligence level because those pedophiles with child sexual offense histories are also more likely to have used child pornography (Seto, 2010). Research is currently focusing on the role of intelligence among pedophilic men who have only consumed child pornography and those who have committed CSA offenses, particularly differentiating those who have been incarcerated from those who have not (Babchishin et al., 2011; Seto et al., 2011, 2012).

As these results indicate, pedophiles do seem to differ from HC on neurodevelopmental measures. However, these results are varied and few strong conclusions can be drawn, including increased rates of left-handedness and increased rates in head injuries before age 13. The next section will discuss the relationship of neurological and neurobiological differences to the development of pedophilia, as both are the focus of current research determining the neural correlates of pedophilia. Please refer to Table 2 for a summarization of neuroimaging findings in pedophilia.

**Table 2:** **Findings from previous neuroimaging studies in pedophilia**.

Author (year)	Method	Structural/functional	PPT groups (***n***)	Paradigm/software	Correction	Threshold/Sig	Findings
Schiffer et al. (2007)	MRI	Frontostriatal and cerebellum structure	Heterosexual (9) and homosexual pedophiles (9) Heterosexual (12) and homosexual (12) controls	VBM-whole brain/SPM 2	FDR (whole brain)/FWE corrected within ROIs	*p* < 0.05	GM volume reductions in pedophiles: PHc L/R, IFG L/R, OFC L/R, Ins L/R, Cer L/R; Cin L/R, Posterior Cin L, STG L/R, MiTG R, Pcu L/R, Put L/R (Amy L/R in unpublished re-analysis)

Schiltz et al. (2007)	MRI	Amygdala structure	Pedophilic (15) Community controls (15)	VBM/manual morphometry/SPM2 ROIs/MRIcro	FWE/corrected for multiple comparisons within ROIs	*p* < 0.05	GM reductions in pedophiles: Amy R, Hyp L/R, SI L/R, Septal Region R, Bed Nucleus Striae Terminalis L/R Enlargement of Temporal Horn R

Poeppl et al. (2013)	MRI	Prefrontal cortex and amygdala structure	Heterosexual (2) and homosexual (7) pedophiles Heterosexual (11) controls	VBM 8 toolbox/SPM 8	FWE corrected within ROIs	*p* < 0.05	GM volume decreases in pedophiles: only in Amy R; pedosexual interest and sexual recidivism associated with GM volume decreases in insular cortex and DLPFC L, preference for younger children associated with GM decreases in the OFC and Ang L/R

Cantor et al. (2008)	MRI	White matter structure	Pedophiles (44) Teleiophilic sexual offenders (21) Non-sexual Offender (53)	VBM whole brain/SPM 2 Parcelation with ANIMAL	FDR	*p* < 0.05	Reduced WM volumes in pedophiles in Superior Fronto-Occipital Fasciculus L, Arcuate Fasciculus R No differences in GM

Cantor and Blanchard (2012)	MRI	White matter structure	Pedophiles (19) Hebephiles (49) Teleiophiles (47)	VBM Whole brain/SPM 2	Not specified	*p* < 0.05	Reduced WM volumes in Temporal Lobe L/R and Parietal Lobe L/R in pedophiles/hebephiles compared to teleiophiles

Cohen et al. (2002)	PET	Frontal and temporal function	Heterosexual pedophiles (7) Community controls (7)	Auditory stimulus/software not specified	Bonferroni	*p* < 0.05	No differences seen in glucose metabolism after an erotic auditory paradigm; lower metabolism in ITC and in Superior VFG during neutral auditory condition in pedophiles compared to controls; no survival after correction

Dressing et al. (2001)	fMRI	Orbitofrontal function	Homosexual pedophiles (1) Controls (2)	Visual stimuli block design/brain voyager	Not specified	Not specified	Stronger recruitment in pedophiles in response to erotic pedohomosexual stimuli: ACC, Brain Stem R, PFC R, Basal Ganglia R, OFC R

Walter et al. (2007)	fMRI	Hypothalamus and lateral prefrontal cortex function	Pedophiles (13) Controls (14)	Visual stimuli/SPM2	Uncorrected	*p* < 0.005	Decreased activations in pedophiles to sexual > emotional arousal contrast: DLPFC R (Precentral), DLPFC R (MFG/SFG), DLPFC L (SFG), Occipital Cortex L

Schiffer et al. (2008a)	fMRI	Frontal and temporal function	Homosexual pedophiles (11) Homosexual matched controls (10)	Visual stimuli/SPM2	Whole brain analysis uncorrected/false discovery rate	*p* < 0.001/p < 0.05	Stronger Activations in pedophiles compared to controls in contrast nude children/adults > dressed children/adults: Fus L/R, HC L/R, Tha R

Schiffer et al. (2008b)	fMRI	Amygdala function	Heterosexual pedophiles (8) Heterosexual matched controls (12)	Visual sexual stimuli/SPM2	Whole brain analysis uncorrected/FDR	*p* < 0.001/p < 0.05	Activations seen in pedophiles compared to controls in contrast nude children/adults > dressed children/adults: MFG R, ACC L/R

Sartorius et al. (2008)	fMRI	Amygdala function	Homosexual pedophiles (10) Heterosexual controls (10)	Visual stimuli/SPM2	Uncorrected	*p* < 0.005	Activation in pedophiles to children (Boys/girls) < neutral geometric stimuli contrasts in Amy R

Poeppl et al. (2011)	fMRI	Cortical and subcortical function	Heterosexual (2) and homosexual (7) pedophiles Heterosexual non-sexual offender controls (11)	Visual sexual stimuli/SPM5	Whole brain analysis uncorrected/FWE/FDR	*p* < 0.001/*p* < 0.05	Activations in pedophiles compared to controls in contrast nude children > scrambled images of children: MFG R, Ins L/R, MTG R, IPL L, Pos R, MCC R, PCC R, HC R, Tha L, Cer R

Ponseti et al. (2012)	fMRI	Pattern classification function	Heterosexual (11) and homosexual (13) pedophiles Heterosexual (18) and homosexual (14) controls	Visual stimuli; pattern classification/SPM8	Uncorrected	*p* < 0.001/*p* < 0.001	Deactivations in homosexual pedophiles compared to controls in boys < men contrast: Cer L/R, Lin L/R, Anterior Tha L, HC R, Occ L, Fus L, ITG R, Ang R Deactivations in heterosexual pedophiles compared to controls in girls < women contrast: NC L/R, SPG L/R, ITG L/R, Fus L/R, Cin L, Occ L, Amy L, Ins L, IFG R, Tha L, Cer R

Habermeyer et al. (2013a)	fMRI	Function	Heterosexual pedophiles (8) Heterosexual controls (8)	Erotic sexual stimuli/brain voyager 2.3.0	Uncorrected/cluster-level threshold correction	*p* < 0.005/*p* < 0.05	Activations in pedophiles in sex × age × group voxel-wise ANOVA analysis in MiFG R

Kärgel et al. (2015)	rsfMRI	Function	Pedophiles + CSA (12) Pedophiles − CSA (14) Healthy Controls (14)	SPM8 and rsfMRI toolkit REST	Uncorrected at voxel level; Family wise error corrected at cluster level	*p* < 0.005/*p* < 0.05	DMN: (P-CSA > P + CSA) Diminished connectivity to left MSF, left OFC. No differences in opposite contrast (P + CSA > P-CSA). (HC > P + CSA): VM PFC, OFC. No differences in P + CSA > HC contrast Limbic Network: (P-CSA > P + CSA) diminished connectivity between L Amy and VM PFC, ACC, OFC, anterior PFC. No differences in P + CSA > P-CSA. In HC > P + CSA contrast: increased connectivity between L Amy and L anterior/inferior PFC, L Lin. No differences in P + CSA > HC contrast

Poeppl et al. (2015)	rsfMRI	Function	Heterosexual (2) and homosexual (7) pedophiles Heterosexual (11) controls	Meta-analytic connectivity modeling (MACM) and ALE	FEW at cluster level	*p* < 0.05	Seed area: R Amy connected to HC, R ventral striatum, R Tha, L Amy, L Cla, L hyp, L Put, L HC, L Mid, L Tha for psychosexual arousal L DLPFC: L Ant Ins, DMPFC, L Per, L SPL, L VLPFC for cognition and perception, spec. working memory L Ins: L PaO, L Ant Ins, L Pos, L STG, L Put, R PaO, R STG, R DLPFC/Ant Ins, R Put, R pMC, L Tha, R Tha, L Ext for perception and cognition

*ACC, anterior cingulate cortex; Amy, amygdala, Ang, angular gyrus, Cau, caudate, CC, corpus callosum; Cer, cerebellum; Cin, cingulate gyrus; Cla, claustrum; DLPFC, dorsolateral prefrontal cortex; Ext, extrastriate cortex; FPPFC, frontopolar prefrontal cortex (Brodmann area 10); Fus, fusiform gyrus; HC, hippocampus; Hyp, hypothalamus; IFG, inferior frontal gyrus; Ins, insula; IPL, inferior parietal lobule; ITC, inferior temporal cortex; ITG, inferior temporal gyrus; L/R, left/right; Lin, lingual gyrus; MCC, middle cingulate cortex; MFG, medial frontal gyrus; MSF, medial superior frontal; Mid, midbrain; MiFG, middle frontal gyrus; MOG, middle occipital gyrus; MTG, middle temporal gyrus; NC, nucleus caudatus; Occ, occipital lobe; OFC, orbitofrontal cortex; PaO, parietal operculum; Par, paracentral lobule; PCC, posterior cingulate cortex; Pcu, precuneus; Per, peristriate cortex; PHc, parahippocampal gyrus; Pos, post central gyrus; Pre, precentral gyrus; PSS, posterior cingulate cortex; Put, putamen; SFG, superior frontal gyrus; SI, substantia innominata; SPG, superior parietal gyrus; SPL, superior parietal lobule; SOG, superior occipital gyrus; STG, superior temporal gyrus; Tha, thalamus; VFG, ventral frontal gyrus*.

### Structural brain alterations in pedophilia

For the purposes of this review, we focused on providing an overview of recent neuroimaging work in pedophilia research starting in 2007, with case studies from 2000 to 2003. This was done for space and readability reasons; such that another recently published review provides an excellent in-depth discussion of neuroimaging in pedophilia (Mohnke et al., 2014). That review summarizes the state of the art of neuroimaging in pedophilia as being in its infancy, with a general consensus that findings are scattered and need to be replicated. Most results from neuroimaging studies in pedophilia have found neurostructural or neurofunctional correlates of CSA, not pedophilia *per se*. The amygdala remains a region of high interest, but Mohnke et al. (2014) suggest stricter methodology to replicate these findings. Our discussion parallels and expands upon the aforementioned review.

A famous case study that highlighted a neurological disease that caused impulsive sexual behavior and could have been an expression of an underlying pedophilic orientation was a right orbitofrontal tumor in a 40-year-old man (Burns and Swerdlow, 2003). Prior to the discovery of his tumor, the patient had overtly claimed no sexual interest in children, but after the tumor progressed, he made sexual advances to his prepubescent stepdaughter and began a pornography collection, including child pornography, resulting from impulse control loss associated with orbitofrontal cortex dysfunction. Although his behavior was non-exclusive and his preference was not explicitly tested, the most striking fact about his symptoms is that all pedophilia-like symptoms disappeared after resection of the tumor. Even more, after the tumor recidivated, the pedophilia-like symptoms remerged and disappeared again after the second resection, thus showing a clear causal link between behavior and brain function. However, the clear majority of orbitofrontal tumors do not result in pedophilic behavior, meaning this case study should be interpreted cautiously.

A further publication with two case studies highlights the role of the temporal cortex in regulating sexual behavior (Mendez et al., 2000). In the first case, a 60-year-old man developed fronto-temporal dementia and presented with increased sexual drive the molestation of extrafamilial children. The second case was a 67-year-old man who developed hippocampal sclerosis that similarly increased his sexual desire. He attempted to molest extrafamilial children. Both patients sexually abused their own young children, suggesting a latent predisposition to pedophilic behaviors existed in these patients prior to disease onset. Both patients showed hypometabolism of the right temporal lobe as measured with FDG-PET. After treatment with antidepressants (paroxetine for the former patient and sertraline for the latter), a decrease in pedophilic behaviors and desires was reported (Mendez et al., 2000). These findings support that dysfunction in the prefrontal cortex may prompt a latent predisposition to sexual attraction to children through disinhibition, whereas a dysfunction in the temporal cortex might elicit this response through sexual preoccupation (Jordan et al., 2011b). This does not explain the etiology of pedophilia as a sexual preference but as an acquired hypersexual behavioral disorder, and furthermore one that rarely presents in the realm of fronto-temporal dementia and hippocampal sclerosis. Clear here is the expression of pedophilic behaviors resulting from the neurological diagnoses, but not why these behaviors were pedophilic rather than hypersexual in nature. For further discussion of dementia and its relation to hypersexual/pedophilic disorders, please refer to Mohnke et al. (2014).

Only a handful of studies of MRI-based structural differences in pedophilia have been published so far. By means of voxel-based morphometry (VBM), several alterations of gray matter (GM) and white matter (WM) were found. In 18 incarcerated exclusive heterosexual and homosexual pedophilic men with histories of sexual offending against prepubertal children, a significantly lower GM volume in the bilateral orbitofrontal cortex, bilateral insula, bilateral ventral striatum (putamen), precuneus, left posterior cingulate, as well as superior and right middle temporal, parahippocampal gyrus, and in the cingulate compared to 24 teleiophiles was found. These findings were corrected for multiple comparisons using the false discovery rate within the whole brain (Schiffer et al., 2007). However, only the left parahippocampal gyrus would have remained significant had a Bonferroni correction for the 15 additional ROI analyses been applied. The authors proposed a theoretical frontal-executive dysfunction and suggested that – similarly to obsessive-compulsive spectrum disorders – these findings may form a neurophysiological circuit contributing to the pathophysiology of pedophilia.

In another study with 15 pedophilic forensic inpatients in comparison to a healthy teleiophile group, GM reductions were found in four pre-defined ROIs comprised of right amygdalae; in right septal region, the bed nucleus striae terminalis (BNST), hypothalamus, and the substantia innominate bilaterally (Schiltz et al., 2007). Later on, amygdalar volume reduction was confirmed by a *post hoc* manual volumetric analysis, unpublished until now (Schiltz, personal communication). These results could be related to a developmental hypoplasia and underscores the influence of right amygdalar lateralization on regulation of sexual behavior, supporting the temporal lobe hypothesis of pedophilia.

One study was published showing that, in comparison to non-sexual offender controls (*n* = 11), convicted pedophilic offenders (*n* = 9) show only GM volume decreases in the centromedial nuclei group of the right amygdala which extended into the laterobasal nuclei group and the cornu ammonis of the hippocampus, although this finding did not survive correction for the large number of predefined ROIs (Poeppl et al., 2013). Interestingly, pedosexual interest, including the strength of such interest, and sexual recidivism were associated with GM volume decreases in the left insular and dorsolateral prefrontal cortices, while preference for younger children was associated with GM decreases in the orbitofrontal cortex and bilateral angular gyri (Poeppl et al., 2013).

What the studies of Schiffer et al. (2007) and Schiltz et al. (2007) have in common is a comparison between a group of sentenced sex offenders recruited from forensic institutions with healthy teleiophiles without criminal histories, leading to potential confounds in the results with factors other than pedophilia, such as criminality or stress of imprisonment. However, an advantage of the study by Schiffer et al. (2007) is that they included only pedophiles of the exclusive type, allowing for interpretations including sexual preference.

By comparing 44 pedophilic men with histories of sexually offending against children or child pornography consumption, with 53 men with histories of non-sexual offenses, differences were found in the WM only, highlighting a bilateral connection route traveling the superior fronto-occipital fasciculus, as well as a right-sided alteration in the arcuate fasciculus. No differences in GM were observed (Cantor et al., 2008). These findings were upheld in a follow-up confirmatory reanalysis (Cantor and Blanchard, 2012) and interpreted as insufficient connectivity in pedophilic individuals, rather than simply GM reductions in disparate (sub-) cortical regions (Cantor and Blanchard, 2012).

Studies to date contain shortcomings either due to the sample sizes, to the configuration of the control group, or because the methodology of VBM was used in a restricted way by focusing on *a priori* regions of interest. The take home message of the present structural imaging MRI studies of pedophilia is that while there have been different results from different studies, one finding has been replicated across studies: reduced right amygdala volumes in pedophiles compared to teleiophilic controls (Mendez et al., 2000; Schiffer et al., 2007; Schiltz et al., 2007; Poeppl et al., 2013). This finding supports the temporal lobe theory of pedophilia referred to in Section “Introduction and Conceptual Framework.” Diffusion-tensor imaging is a method of WM imaging that holds promise to validate and expand previous VBM results.

### Functional brain alterations in pedophilia

Only a few functional imaging studies have been conducted to investigate possible differences during the processing of sexual stimuli in the brains of pedophiles. With only one exception, they were visual sexual stimulation studies, thereby inducing a strong visual bias while making this modality the dominant model of perceptual processing alterations in paraphilias, although sensory systems offer potential other routes to sexual responsiveness. However, with the background of recent evidence explaining how hetero- or homosexual teleiophilic brains process visual sexual information and regulate the psychosexual and physiosexual components of sexual arousal [please refer to Safron et al. (2007), Georgiadis and Kringelbach (2012), Stoléru et al. (2012), and Poeppl et al. (2014)] for a deeper discussion), it is a reasonable step toward the understanding of pedophilia to study whether there are functional differences in the brain network associated with sexually arousing visual pictures of children.

Research has highlighted alterations in pedophiles through positron emission tomography (PET) and functional MRI. For example, in a PET study of pedophilia, a decreased regional cerebral metabolic rate for glucose was found in the right inferior temporal cortex and superior frontal gyrus, without Bonferroni correction. This rate decreased in the pedophilic group after presentation of girl and women cues, whereas it increased in the teleiophilic group (Cohen et al., 2002). The authors interpreted this as a consistent brain abnormality underlying decreased glucose metabolism in the temporal and frontal cortices implicated in cortical regulation of sexual arousal. The small sample size of seven participants in each group limits the generalizability and confidence with which the results can be interpreted.

In fMRI research, the first study that included a single homosexual pedophile found increased activity of the anterior cingulate gyrus, right prefrontal cortex, and basal ganglia in response to pictures of minimally clothed boys, regions that comprise the attentional brain network with the right orbitofrontal cortex (Dressing et al., 2001).

Decreased activations were seen in the hypothalamus, dorsal midbrain, dorsolateral prefrontal cortex, and right lateral parietal, right ventrolateral, and right occipital cortices, as well as in the left insula in 13 hetero- and homosexual forensic pedophiles when viewing sexual stimuli as compared to emotional stimuli as compared to teleiophiles (Walter et al., 2007). Based on these findings, it was suggested that the missing sexual interest toward adults could be explained by impairment in subcortical regions associated with the autonomic component of sexual arousal, i.e., lack of activation seen in hypothalamus and dorsal midbrain in pedophilia. Additionally, using a regression analysis approach, the activation in the left DLPFC was inversely correlated with the score on the child abuse subscale of the multiphasic sexual inventory (MSI), indicating also possible alterations of cognitive processing of sexual stimuli in these subjects (Schiffer et al., 2008a,b).

Homosexual and heterosexual incarcerated pedophiles were examined with fMRI to determine whether there were differences associated with age and child gender preference. Among homosexual pedophiles with a history of sexual offenses against children (*n* = 11) in comparison with homosexual (*n* = 12) controls, the substantia nigra, caudate nucleus, the occipitotemporal and prefrontal cortices, thalamus, globus pallidus, and the striatum were activated in response to male child sexual stimuli, whereas these were not among the matched homosexual teleiophiles (Schiffer et al., 2008a). This was interpreted as an increased effort in evaluating respective stimuli in pedophilic compared to control participants. In another investigation, heterosexual pedophiles (*n* = 8), when compared to heterosexual teleiophiles (*n* = 12), after presentation with female child sexual stimuli displayed significant activations in the amygdala, hippocampus, substantia nigra, caudate nucleus, the medial dorsal thalamic nucleus, and the inferior temporal gyrus, suggesting a similar response pattern to sexually preferred stimuli as seen in healthy heterosexual males (Schiffer et al., 2008b). Pedophilic males showed a signal increase only in the right dorsolateral prefrontal cortex in response to the preferred sexual stimuli (no activation was seen in the control group to sexual stimuli of adult women). An interesting finding was that whereas the healthy male teleiophiles activated the orbitofrontal cortex in response to both sexually explicit adult female and female child imagery, this activation was not seen among male pedophiles. All together, the authors suggest that orbitofrontal deactivation, as shown in pedophilic participants, represents a dysfunction of the neural network necessary for the appropriate cognitive component of sexual arousal processing.

There were also attempts to investigate the perception and emotional processing of visual sexual stimuli. For example, the right amygdala showed greater activation in homosexual pedophiles when they were presented with male child sexual stimuli compared to heterosexual male teleiophiles who observed female adult sexual pictures, although the participants were not matched for sexual orientation, thus potentially obscuring true ‘pedophilic’ activations (Sartorius et al., 2008). The authors interpreted this increased amygdala activation to stimuli depicting children that were observed in pedophiles as a possible fearful emotional reaction combined with sexual arousal, supported by the lack of an appropriate amygdala activation to adult female stimuli (Sartorius et al., 2008).

Poeppl et al. (2011) used a block design in their study to investigate sexual interest in pedophiles (nine pedophiles with a history of contact offenses and 11 non-sexual offender controls) that consisted of male and female nude Tanner scale imagery, including Tanner scales I, III, and V, corresponding to prepubescent, pubescent, and adult images. Results of whole brain analyses showed significantly greater activation in the middle temporal lobe, hippocampus, posterior cingulate cortex, thalamus, medial frontal lobe, and culmen of the cerebellum in pedophiles to the Tanner I > neutral contrast. When compared to control teleiophiles in the Tanner V > neutral contrast, pedophiles showed a significant deactivation in the right insula. Furthermore, in the between group contrast of interest (pedophiles > Tanner I, teleiophiles > Tanner V), there were significantly greater activation signals seen in the postcentral gyrus, right middle temporal gyrus, anterior midcingulate cortex, and the amygdalae bilaterally (Poeppl et al., 2011). The authors interpreted these findings as an easier sexual arousability in pedophilic as compared to non-paraphilic participants when stimulated with purposefully non-erotic material (Poeppl et al., 2011).

In a similar study, Habermeyer et al. (2013a) investigated eight pedophiles (three with a history of contact offenses, five with a history of child pornography consumption) and eight heterosexual teleiophilic controls in an event-related design consisting of erotic pictures of boys, girls, men, and women. In an ROI analysis including the middle frontal gyrus, only the pedophilic participants showed activation in the girl contrast, whereas controls showed deactivation (Habermeyer et al., 2013a). A further finding showed that during the immediate processing of erotic stimuli, both groups showed significant activations in the dorsomedial prefrontal cortex, a finding the authors attributed to the crucial role this region occupies in the critical evaluation of and attention to sexual stimuli (Habermeyer et al., 2013a).

Two recent studies investigated functional connectivity in pedophilia and have supported decreased connectivity associated with CSA, but not with pedophilia. Specifically, Kärgel et al. (2015) examined functional connectivity at rest (RSFC) in 26 pedophilic men stratified according to offense status (14 P+CSA, 12 P–CSA) and 14 HC within (1) the default mode network and (2) the limbic network. Pedophiles who engaged in CSA depicted diminished RSFC in both networks compared with HC and P–CSA with diminished RSFC between the left amygdala and orbitofrontal as well as anterior prefrontal regions. These findings highlight a diminished resting state functional connectivity in offending pedophiles as compared to controls, suggesting a relationship to CSA more than to pedophilia. Using complex multimodal integration of brain structure and function analyses, Poeppl et al. (2015) found that the functional role of brain regions that are altered in pedophilia were linked to non-sexual emotional as well as neurocognitive and executive functions, which were previously reported to be impaired in pedophiles. They suggested that structural brain alterations affect neural networks for sexual processing by way of disrupted functional connectivity and that structural alterations also account for common affective and neurocognitive impairments in pedophilia.

Further, new methods have been investigating differences that go beyond regional activations. Pattern classification is a new method of analyzing neural activation patterns. The idea of pattern classification is to use activation patterns in different brain regions in a multivariate approach rather than relying on region by region comparisons (Linden, 2012). It can be used for classifying groups. For example, in the field of sexology pattern classification has been applied successfully to classify heterosexual and homosexual male teleiophiles (Ponseti et al., 2009).

**Figure 3:**
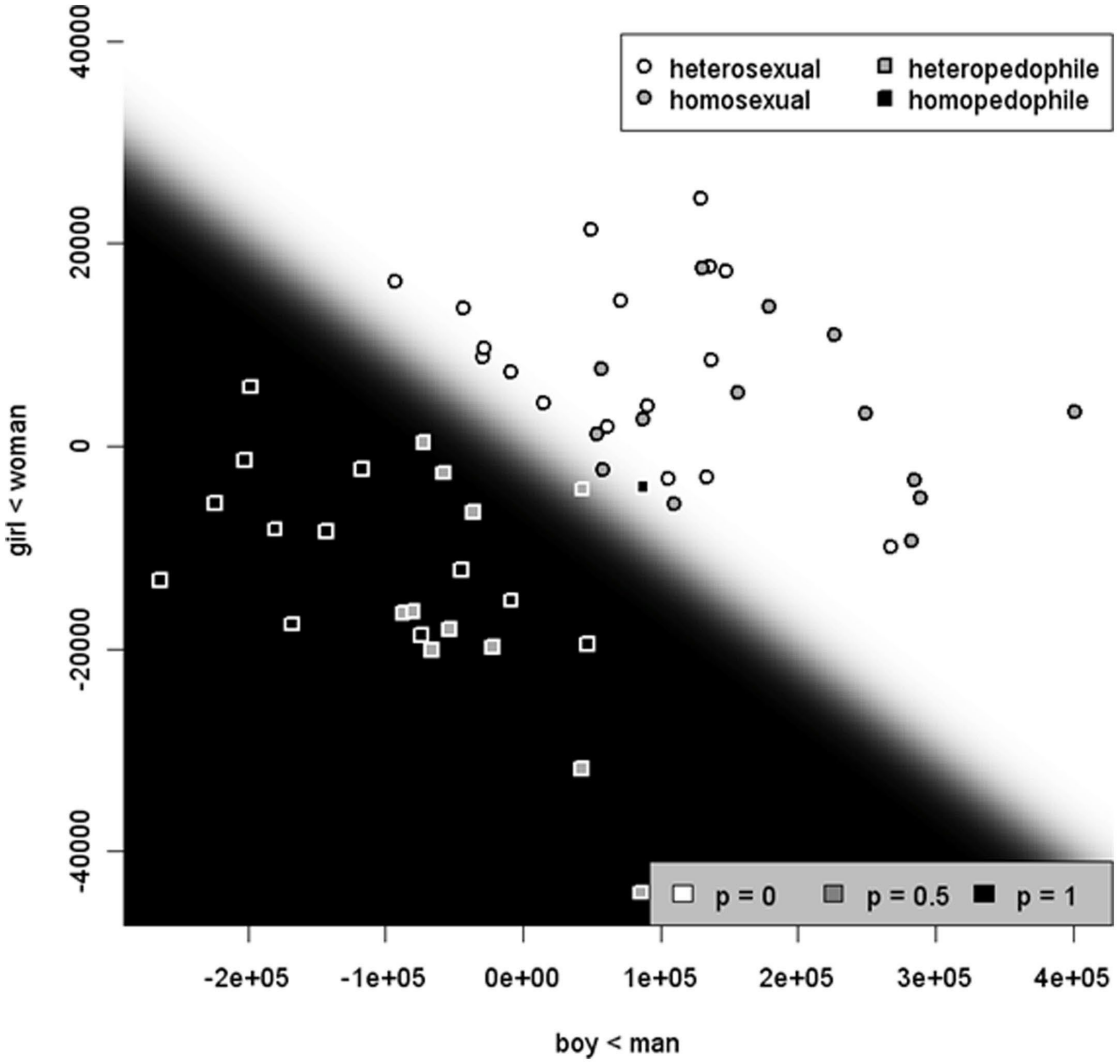
**Illustration of pattern classification of pedophiles and healthy controls using individual expression values**. Participants with p >0.5 (dark area) were classified as pedophiles. For further details, see Ponseti et al. (2012).

Research found that the activations seen in heterosexual and homosexual pedophiles to child stimuli are nearly indistinguishable from those in heterosexual and homosexual healthy males to adult stimuli (Ponseti et al., 2012); this supports the assumption that pedophilia is primarily a sexual age preference similarly to teleiophilia. The activation pattern among heterosexual and homosexual pedophiles and healthy male teleiophiles includes the caudate nucleus, cingulate cortex, insula, fusiform gyrus, temporal cortex, occipital cortex, thalamus, amygdala, and cerebellum. Despite the similarity in activation patterns between pedophilic and teleiophilic men, the novel pattern classification technique has been successfully applied based on the presentation of preferred sexual stimuli and resulted in a mean accuracy of 95%, with 100% specificity and 88% sensitivity (Ponseti et al., 2012; Mohnke et al., 2014), thereby showing a promising new approach for classifying subjects. Please refer to Figure 3 for a visual explanation of pattern classification according to Ponseti et al. (2012). These studies included fully admitting pedophilic participants only; therefore, further research should verify its use with partially- or non-admitting pedophiles. The promise of functional predictors is, however, also supported by a similar study which, in contrast to Ponseti et al. (2012), used a highly hypothesis-driven approach of several impaired functions.

In their study, Walter et al. (2010) could show that a bi-dimensional discriminant function analysis revealed highly significant group separation when activations for cognitive appraisal or passive consumption of visual material are considered in their respective specific brain regions. A recent study investigated response inhibition in pedophilic males and found that pedophilic participants had slower reaction times and less accurate visual target discrimination which corresponded to greater activation in the “No-Go” condition for the DLPFC bilaterally, frontal eye fields, and supplementary motor areas, but in the left anterior cingulate cortex, precuneus, and angular gyrus, they showed greater activation in the “Go” condition (Habermeyer et al., 2013b) in an uncorrected voxel-wise analysis.

As research shows, there are regions that differ in neural activation among heterosexual pedophiles, homosexual pedophiles, and matched healthy teleiophiles. However, limitations in these early studies included controlling neither for sexual preference nor orientation, using insufficiently differentiated inter-study paradigms such that all generalizations had to be limited to the exact study and paradigm utilized. Furthermore, pedophilic participants were all incarcerated or judicially involved [a notable exclusion to this being (Ponseti et al., 2012)], underscoring the need for studies investigating non-incarcerated pedophilic participants. As previous research in normal human sexuality has shown, there are notable differences between healthy heterosexual and homosexual men. This should be kept in mind for future neuroscientific investigations (Hamann et al., 2004; Ponseti et al., 2006, 2009).

As discussed previously, neuroimaging is a useful way of investigating the neural correlates of human sexuality in terms of detecting the arousal pattern associated to the sexual preference structure. A criticism of previous functional neuroimaging studies in pedophilia relates to faking. Under the assumption that immediate processing of sexual stimuli is outside of conscious cognitive control (bottom-up influence), results were interpreted so that (de)-activations were true and not the result of faking (Ponseti et al., 2009, 2012, 2014). However, studies of test-retest reliabilities and faking in fMRI research have shown that faking can and does occur (Lee et al., 2009) and that, findings are not always reliable across centers and studies (Maitra et al., 2002; Raemaekers et al., 2007; Friedman et al., 2008). With the aforementioned limitations in mind, new research programs will help to differentiate the true differences from methodological artifacts.

### The contribution of molecular genetics and epigenetics

Even though first hints for a familial transmission of pedophilia date back to the early 80s (Gaffney et al., 1984), only limited research has been conducted into the genetic contributions to pedophilia. Twin studies of sexual orientation hint at a heritable component of homosexuality (Bailey et al., 2000; Santtila et al., 2008; Långström et al., 2010). Most recently, a Finnish group published the first twin study investigating pedophilia. This was a population-based twin design analyzing 3967 male twins and their male siblings. It was shown that genetic influences contribute to sexual interest, fantasies, or activity pertaining to children under the age of 16 years (Alanko et al., 2010). However, the heritability estimated in the study explained only 14.6% of the variance; in comparison, the heritability of almost all psychiatric disorders is estimated to be above 30%, with schizophrenia and bipolar disorder ranging as high as 70–80% (Alanko et al., 2013). Based on their findings, the authors concluded that future research should address the possible interplay of genetic with environmental risk factors, such as own sexual victimization in childhood (Bienvenu et al., 2011). Another recently published study reported genograms of five families with unusual high occurrence of paraphilias (mainly pedophilia). They found familial aggregation of paraphilias with no clear Mendelian type of transmission. Intriguingly these families included carriers for different types of possible developmental disorders such as conduct disorders, deafness, blindness, and epilepsy (Labelle et al., 2012).

No candidate studies nor genome-wide association studies in the field of pedophilia have been published today and to our knowledge, no large-scale efforts to fill this gap are currently under way. This would also not be feasible considering that the number of subjects needed in order to expect genome-wide significant findings would be in the range of several thousand or ten-thousand.

Given the weak heritability of pedophilia together with the assumed large effects of early environment and early development, and possibly an interaction among these different factors, epigenetics might represent a promising way to disentangle the biological substrates and possible markers of sexual deviation. Epigenetics is the study of the dynamic changes in gene regulation, which the organism achieves using the common mechanisms of DNA methylation, histone modification, and chromatine restructuring (Rodenhiser and Mann, 2006). Through epigenetic mechanisms, the organism can establish a molecular memory of past gene × environment interactions, with long-lasting effects on brain circuits and genetic pathways. For example, early life stress programs the function of the HPA-axis through epigenetic alterations in the regulation of key genes involved in HPA axis functioning (Szyf et al., 2005; McGowan et al., 2009; Muragtroyd et al., 2009). Epigenetic (dys-) regulation plays an important role in different neuropsychiatric disorders and was proven as a successful heuristic framework for research in neurodevelopmental disorders (Krebs et al., 2004; Schroeder et al., 2012). Epigenetic mechanisms are also involved in the process of tissue differentiation (Rodenhiser and Mann, 2006) as well as in normal sexual dimorphic brain development (Nugent et al., 2011). Recent findings give rise to the view that epigenetic mechanisms are at the core of sexual differentiation and serve as the interface between hormonally transmitted changes and sex chromosome related effects (Arnold et al., 2012). Its implication in both normal and abnormal brain development, as well as its role in the etiology of psychiatric disorders, makes it likely that epigenetic mechanisms widely contribute to the development of the human sexual preference structure including pedophilia. However, to date, no investigations of epigenetics in this direction have been published.

## Conclusion: What are the Implications and Future Directions of Neurobiology and Pedophilia?

Previous research investigated the etiology of pedophilia from a neurobiological and neurodevelopmental perspective, utilizing state-of-the-art neuroimaging equipment and methods and physical markers known to be highly influenced by developmental challenges. Although the idea of a neurodevelopmental etiology of pedophilia has a very wide scope and this idea can be attributed to other psychological disorders, we feel its relationship to pedophilia warrants stricter research.

Support for a neurodevelopmental pathway comes from research investigating epigenetic dysregulation of sexual development in general, physical characteristics, and functional as well as structural brain differences in pedophilia. Pedophilia seems to have a small hereditary component, with cases clustering in families and familial transmission of deviant sexual fantasies and behaviors (Gaffney et al., 1984; Alanko et al., 2010).

Sexually offending and incarcerated pedophilic men show increased rates of left-handedness, have shorter stature, experience twice as many head injuries before the age of 13 as normal counterparts, and seem to have lower intelligence than teleiophilic men (Blanchard et al., 2003, 2007; Cantor et al., 2004, 2005, 2007). These variables are present in pedophilic men significantly more often than in healthy control, but it is not clear if the reason for this is the sexual behavior disorder, the pedophilic preference, or even another factor.

The push for neurobiological research has resulted in three major aforementioned theoretical developments, all attempting to explain various aspects of pedophilia. The frontal lobe theory is a contender to explain offenses against children from behavioral disinhibition and uncontrolled compulsive behaviors. Noticeable structural and functional differences in size and function of the left and right dorsolateral prefrontal and orbitofrontal cortex have been found in pedophilic men with a history of contact sexual offenses against children (Burns and Swerdlow, 2003; Schiffer et al., 2007, 2008a,b; Poeppl et al., 2011).

The temporal-limbic theory tries to explain pedophilia through structural and functional differences in the temporal lobes, thus focusing on the misattributed emotional salience and valence toward children. Several case studies highlight temporal and amygdalar lesions or functional activation differences that might contribute to the development of a pedophilic sexual preference (Cohen et al., 2002; Joyal et al., 2007; Schiltz et al., 2007; Walter et al., 2007; Sartorius et al., 2008).

The dual lobe theory suggests that both frontal and temporal disturbances are responsible for the range of behaviors seen in pedophilia, such as diminished impulse control as seen with orbitofrontal deficits and hypersexuality through the temporal lobes (Seto, 2008, 2009; Poeppl et al., 2013).

**Figure 4:**
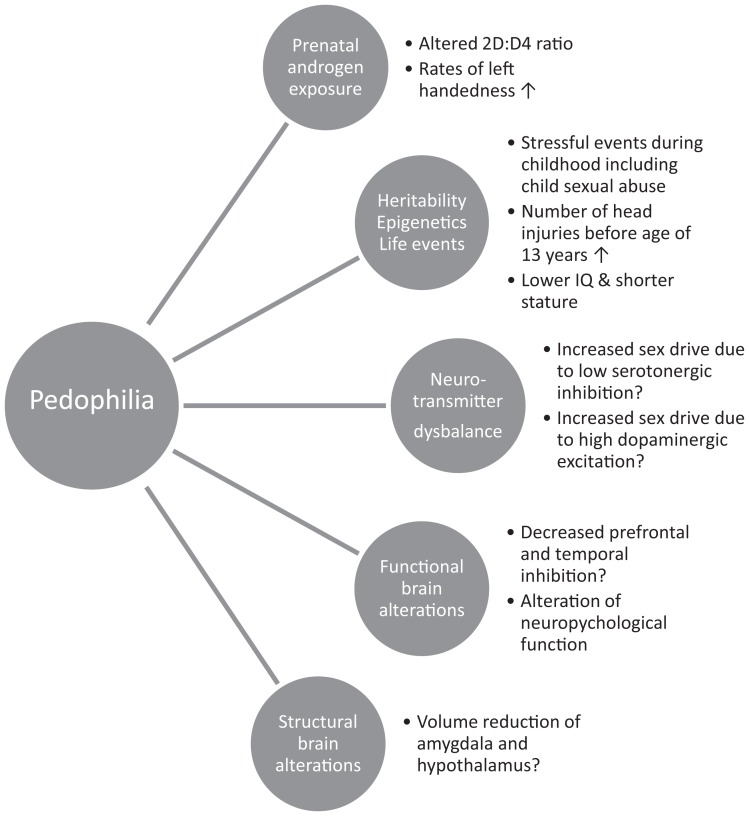
**Findings and questions regarding the etiology of pedophilia**.

Therefore, future investigations in the neuroimaging of pedophilia should use stricter inclusion/exclusion criteria to better limit potential confounds and actively recruit non-offending pedophiles to close the gap in knowledge between offending and non-offending pedophiles. This will also aid in researchers’ abilities to understand exactly what regions of the brain are implicated in pedophilic sexual preference development, as current literature interpretation implicates the brain in an overly ambitious manner. Examinations of the symptomatology and clinical aspects of pedophilia should first try to replicate original findings before novel ideas can be properly tested, including testosterone and its role in pedophilia development or the role of neurotransmitters such as dopamine and serotonin and their receptor densities in relation to behavioral perturbations. What is ultimately needed in this research field are stricter participant inclusion criteria and studies utilizing non-offending pedophiles and non-pedophilic offenders in order to ascertain what differences are true to pedophilia and those that are true to sexual offending against children in general. Please refer to Figure 4 for a visual of research questions and directions for the etiology and treatment of pedophilia.

Now that pedophilia is an increasingly accepted research field and not only a side issue, scientists are more intensively investigating not only how it develops, but also how to treat, and ultimately, how to prevent offending against children. Ultimately, the success rests with researchers willing to investigate a topic that still carries a significant societal stigma load but promises to offer a significant improvement not only to patients but also to society in general.

## Conflict of Interest Statement

The authors declare that the research was conducted in the absence of any commercial or financial relationships that could be construed as a potential conflict of interest. The Reviewer Stuart Brody declares that, despite having collaborated with the author Tillmann H. C. Kruger, the review process was handled objectively.
